# Long-term atorvastatin improves cognitive decline by regulating gut function in naturally ageing rats

**DOI:** 10.1186/s12979-022-00311-x

**Published:** 2022-11-09

**Authors:** Tian-Ce Xu, Yan Lv, Quan-Ying Liu, Hui-Sheng Chen

**Affiliations:** Department of Neurology, General Hospital of Northern Theater Command, Shenyang, 110016 China

**Keywords:** Gut microbiota, Atorvastatin, Gut-brain axis, Ageing, Cognitive decline

## Abstract

**Background:**

Statins have been widely used to prevent cardiovascular disease in middle-aged and elderly populations; however, the effect of long-term treatment on cognitive function is controversial. To simulate clinical conditions, middle-aged rats were given atorvastatin for 9 consecutive months to investigate the effect on natural cognitive decline and the possible mechanisms.

**Results:**

The results showed that compared with the control group, long-term atorvastatin treatment naturally improved cognitive decline. Furthermore, long-term treatment regulated intestinal retinoic acid (RA) metabolism and storage by altering retinol dehydrogenase 7 (Rdh7) expression in the intestine, while RA metabolism affected the proliferation of intestinal T_reg_ cells and inhibited IL-17^+^γδ T-cell function. In addition, long-term atorvastatin increased intestinal flora richness and decreased IL-17 expression in hippocampal tissue.

**Conclusion:**

Collectively, these findings provide the first evidence that long-term atorvastatin intervention may prevent cognitive decline in naturally ageing rats by inhibiting neuroinflammation via the gut-brain axis.

## Background

With increasing age, people's physiological functions gradually deteriorate, and a decrease in cognition is one of the most obvious characteristics of ageing [1]. Therefore, preventing and improving the degradation of cognitive function in the process of ageing is a great challenge in modern ageing society.

Accumulating studies have demonstrated the importance of the brain-gut axis [2, 3], where the inflammatory process should play a key role. Several recent studies have confirmed that intestinal flora, brain inflammation, and cerebral or cognitive impairment are closely related [4–6]. As a metabolite of vitamin A, retinoic acid (RA) was found to regulate the transcriptional process, affect the differentiation and function of B cells, T cells, and bone marrow cells, and drive intestinal protective or pathogenic immune responses in a concentration-dependent manner [7]. At high concentrations, RA promotes the differentiation of naive T cells into T_reg_ cells, which play an integral role in maintaining the anti-inflammatory environment in the intestine by inhibiting T helper (Th)17 cell differentiation and γδ T-cell proliferation [8–10]. At low concentrations, RA is essential for the production of the proinflammatory cytokines interferon (IFN)-γ and IL-17A by Th1 and Th17 cells to respond to infection and coordinate the inflammatory immune response [11–15].

Statins, as effective medications for cardiovascular disease, are one of the most prescribed drugs in the world [16]. Some studies have observed potential adverse effects of statins in patients with normal cognition and cognitive impairment [17, 18]. However, there is growing evidence that statins may have a protective effect on dementia or cognitive function [19–22]. For example, a meta-analysis found that statins reduced the relative risk of developing systemic dementia [23], and a higher concentration of low-density lipoprotein cholesterol (LDL-C) was associated with a higher risk of Alzheimer’s disease (AD) [24]. It is worth noting that the positive effect of statins on cognitive function may have nothing to do with cholesterol levels [25]. Collectively, there should exist other mechanisms underlying the effect of statins on cognition in addition to lipid-lowering effects.

As a widely used statin, atorvastatin has been found to reduce neuroinflammatory damage and improve cognitive function in AD mice [26]. However, whether atorvastatin can reduce neuroinflammation via the gut-brain axis is still unclear. In this context, we hypothesize that long-term statin use can exert neuroprotective effects on cognitive function via the brain-gut axis. In the current study, we will explore (1) whether long-term atorvastatin has an effect on cognitive function in naturally ageing rats and (2) whether the brain-gut axis is involved in the mechanisms underlying the effect.

## Methods

### Experimental model and subject details

Adult male SD rats (SPF level, 9 months of age) were housed in an environment with free access to food and water, a 12-h light/12-h dark cycle, and a temperature of 24 ± °C. These experiments were approved by the Animal Care and Use Committee of the General Hospital of Northern Theater Command and conformed to the principles outlined in the National Institutes of Health guidelines. The number of animals used at each step was minimized.

A total of 30 rats were used in this study. They were randomly divided into 3 groups of 10 rats each. The groups were as follows: (1) control group: intragastric administration of 0.5 ml saline each day for 9 consecutive months; (2) low-dose group: intragastric administration of atorvastatin (2.1 mg/kg, dissolved in 0.5 ml saline) each day for 9 consecutive months; (3) high-dose group: intragastric administration of atorvastatin (8.4 mg/kg, dissolved in 0.5 ml saline) each day for 9 consecutive months. Atorvastatin is a gift from Pfizer (Pfizer Inc.). Assuming a human body weight of 60 kg, the drug dose is based on the low dose (20 mg/d) and the high dose (80 mg/d). According to the equivalent dose conversion method for experimental animals, the conversion factor for SD rats was 6.3, and therefore the low dose for SD rats was 2.1 mg/kg/d and the high dose was 8.4 mg/kg/d. Investigators were blinded to group allocation during the animal experiments.

## Method details

### Y-maze

The Y-maze consists of three equally angled arms (30 × 5 × 12 cm). The rat was placed at the end of one arm and allowed to move freely through the maze for 6 min. The rats were counted when their hind paws were completely inside the arm, and the percentage of alternation was calculated. Spontaneous alternation was defined as successful entry into three sectors. The percentage of alternation was calculated as the ratio of actual rotations to possible rotations (defined as the total number of arms entered minus two). Percentage of alternation = [Number of corrects/(Total number of arm entries—2)] × 100%.

### Novel object recognition

Two identical cylinders (object A, old object) were placed in the rat cage for five minutes during the adaptation phase. The rats actively explored the two objects due to their curiosity about novelty. The rats rested for 1 h and then performed the test phase. One of the A objects was removed and replaced with a cube (object B, new object). After placing and immediately turning on the video equipment, the experimenter immediately left the test room and recorded the rat's contact with the two objects, including the number of times the nose or mouth touched the object and the time spent exploring within 2–3 cm from the object (front paws on the object, nose sniffing the object, licking the object). The time the rats spent exploring objects A and B was recorded. The discrimination index = object B/(object B + object A) × 100%.

### Morris water maze

The Morris water maze consists of a circular pool, an underwater platform, and an automatic image acquisition and processing system (camera, video recorder, monitor, and analysis software). In this experiment, the Morris water maze is a circular pool with a diameter of 150 cm and a height of 40 cm. The interior of the pool is black, the water depth is 24 cm, and the water temperature is kept at (22 ± 2) °C. The light in the room was constant, with no direct light in the pool. The pool was divided into four quadrants with four equidistant points on the pool wall. In the target quadrant (set as quadrant 1), a circular station with a diameter of 12 cm and a height of 23 cm was placed at a distance of 30 cm from the pool wall, and the station was located 1 cm below the water surface. A camera connected to the display system was placed above the labyrinth. The Morris water maze video analysis system was used for information processing. Procedure and platform quadrant setting: The experiment used the classical Morris water-maze test procedure, and the experiment was conducted for 6 d. The first 5 d was for the positioning navigation test, and the last 1 d was for the space exploration test. The platform was set in quadrant 1, and its opposite side was quadrant 3. The quadrants were set in the following order: quadrant 1, quadrant 2, quadrant 3, and quadrant 4.

Positioning navigation test: The rat was placed in the pool from quadrant 1 for a maximum recording time of 90 s. If the rat could not climb on the platform within 90 s, it was guided to climb on the platform for 10 s and finally dried and placed into the cage. In this way, the test was conducted in quadrant 1 and then in quadrant 2, quadrant 3, and quadrant 4 for 4 quadrants per day for 5 d. The average latency to climb on the platform in each of the 4 quadrants was recorded to evaluate the spatial learning ability of the mice.

Spatial exploration test: On Day 6 of the experiment, the environment and water temperature were the same as those in the positioning navigation test. The platform under the water surface was removed, and then the rat was placed into the pool from quadrant 3. The swimming trajectory of the rat in 90 s was recorded and analysed. The number of times the rat crossed the platform and the percentage of time spent in quadrant 1 (target quadrant) were recorded to judge the spatial memory ability of the rat.

### LC‒MS

All solvents and reagents were chromatographic grade, and retinoic acid, retinol, and retinyl acetate standards were purchased from Shanghai Yuan ye Biotechnology Company. The samples were rat intestinal tissue samples from each experimental group. In brief, 50 mg of tissue was homogenized with 1 ml of n-hexane, and samples were subsequently spun at 12,000 rpm for 10 min at 4 °C. Then, 500 μl of supernatant was collected and evaporated to dryness at room temperature. The residue was resuspended in 200 μl MeOH. After centrifugation at 12,000 rpm for 10 min at 4 °C, 10 μl supernatant was transferred to LC vials containing glass inserts for analysis. The instrument used for LC‒MS analysis was a Shimadzu ultrahigh-performance liquid chromatograph (LC-30AT) connected to a SCIEX 5600 + mass spectrometer. The chromatographic column was an X Bridge BEH C18 column, 130 Å, 2.5 µm, 2.1 mm X 150 mm, and the mobile phases were A: 0.1% formic acid–water and B: 0.1% formic acid-acetonitrile, with gradient elution from 50% A to 0% A for 0–7 min and 0% A for 7–15 min. The elution flow rate was 0.3 mL/min. The column temperature was 40 °C, and the injector temperature was 15 °C. The mass spectrometry scan was performed in positive mode.

### Cell isolation from intestine tissue

The small intestines were removed and cleaned of fat and faeces, the Peyer’s patches were removed, and the intestines were subsequently washed twice with D-Hank’s solution containing antibiotics. The intestines were divided into 1 cm pieces and incubated in shock with 20 ml Hanks’ balanced salt solution (HBSS) containing 10 mM HEPES, 4 mM EDTA, 8% FBS and 0.5 mM DTT for 20 min at 37 °C. After incubation, the samples were vortexed, small intestinal intraepithelial lymphocytes (IELs) were collected from the solution through a 70 μm cell strainer, and the tissue was reincubated as above. The remaining tissue was washed in PBS and further incubated with 5 ml of HBSS digestive solution (containing 10 mM HEPES, 5% FBS and 0.2 mg/ml collagenase D) for 20 min at 37 °C. The samples were subsequently vortexed, and small intestinal lamina propria mononuclear cells (LPMCs) were collected through a 40 μm cell strainer.

The obtained IELs and LPMCs were centrifuged at 500 g at 4 °C for 10 min, 8 ml of 44% Percoll lymphocyte isolate was added, and the cells were resuspended and spread flat on top of 5 ml of 67% Percoll lymphocyte isolate, keeping the interface between the two layers of Percoll lymphocyte isolate clear. After centrifugation at 500 × g for 20 min at 4 °C, a cloudy cell layer was visible between the 44% Percoll lymphocyte isolate and the 67% Percoll lymphocyte isolate, and the cells between the 44% and 67% Percoll interfaces were collected.

### Flow cytometric analysis

The cell concentration was adjusted to 1 × 10^6^/ml by mixing. The above cell suspensions were incubated with CD4 and CD45 antibodies or CD45 and TCR-γδ antibodies for 30 min. After washing with PBS containing 3% foetal bovine serum. The cells were incubated overnight at 4 °C in fixation/permeabilization solution, Foxp3 antibody or IL-17 antibody was added, and the cells were incubated for 30 min, fixed with 1% paraformaldehyde PBS solution and then placed on the machine to be measured.

### Immunofluorescence

Small intestinal Sects. (5 μm) were deparaffinized and rehydrated, and antigen retrieval was performed (with citrate buffer). Sections were blocked in goat serum for 15 min and washed three times with PBS. Slides were subsequently incubated overnight at 4 °C with antibodies against Rdh7. Thereafter, the slides were incubated with a secondary antibody conjugated to Cy3. After washing 3 times, nuclei were stained with DAPI. Mounted sections were observed and photographed by fluorescence microscopy. The experimental method for neurofibrillary tangles of brain tissue was similar to the previous method. P-Tau^S202/T205^ (green fluorescence) can be used to localize neurofibrillary tangles [27].

## RT‒PCR

Total RNA was extracted from tissue samples according to the TRIzol kit and then reverse transcribed into cDNA after identification and purification. The cDNA template (1 μl), upstream primer (0.5 μl), downstream primer (0.5 μl), and SYBR GREEN master mix (10 μl) were added to the PCR tube in order, and the total volume was adjusted to 20 μl with double distilled water. Fluorescence quantitative PCR conditions: 95 °C for 3 min; 94 °C for 15 s, 56 °C for 30 s, 70 °C for 60 s, 35 cycles; 4 °C for 5 min. The experimental results were analysed using a fluorescence quantitative analyser, and the Ct values were converted to the corresponding values using the 2^−△△Ct^ method for quantitative analysis of the target genes.

### Western blot

The hippocampus was extracted using a Whole Cell Lysis Assay kit. Protein samples were quantified using a BCA kit, separated by SDS‒PAGE, transferred to transwell transfer packs and blocked for 1 h with 5% dry milk in 0.15% Tween-20 in TBS (TBST buffer). The membranes were then washed and incubated with primary antibody overnight. The secondary antibody was added and incubated at room temperature for 2 h. After washing 4 times with TBST buffer, immunoreactive bands were visualized by chemiluminescent detection (ECL). The optical density value of the target strips was analysed by Gel-Pro-Analyser software.

### 16S rRNA microbiome sequencing and microbial community analysis

The genomic DNA of the samples was extracted using the SDS method, followed by agarose gel electrophoresis to detect the purity and concentration of DNA. An appropriate amount of sample DNA was placed in a centrifuge tube, and the sample was diluted to 1 ng/μl using sterile water. PCR was performed using diluted genomic DNA as a template, specific primers with Barcode according to the selection of the sequencing region, Phusion® High-Fidelity PCR Master Mix with GC Buffer from New England Biolabs, and high-efficiency high-fidelity enzymes to ensure amplification efficiency and accuracy.

The data of each sample were split from the downstream data according to the barcode sequence and PCR amplification primer sequence, and the reads of each sample were spliced using FLASH (V1.2.7) [28] after truncating the barcode and primer sequences. The obtained spliced sequences are raw tags; the raw tags obtained by splicing need to go through a strict filtering process [29]to obtain clean tags.

Observed-otus, ace, and PD whole tree indices were calculated using Qiime software (Version 1.9.1). Dilution curves, rank abundance curves, and species accumulation curves were plotted using R software (Version 2.15.3). The analysis of variance between groups of alpha diversity index was performed using R software; the analysis of variance between groups of alpha diversity index will be performed with and without parametric tests, respectively, and the Tukey test and the Wilcoxon test were chosen. LDA effect size analysis using LDA effect size software (Figure by Figdraw).

### Quantification and statistical analysis

Statistical analysis is described in each figure legend. Data were analysed using Prism software (GraphPad 8.0). Data are expressed as the mean ± SEM. Differences between the groups in the behavioural experiments were analysed by repeated-measure ANOVA. One-way ANOVA was used for multiple group comparisons. *p* values < 0.05 were considered significant, < 0.01 were considered very significant, and < 0.001 were considered highly significant.

## Results

### Long-term atorvastatin improved cognitive function but had no effect on *pancreatic islet function*

**Fig. 1:**
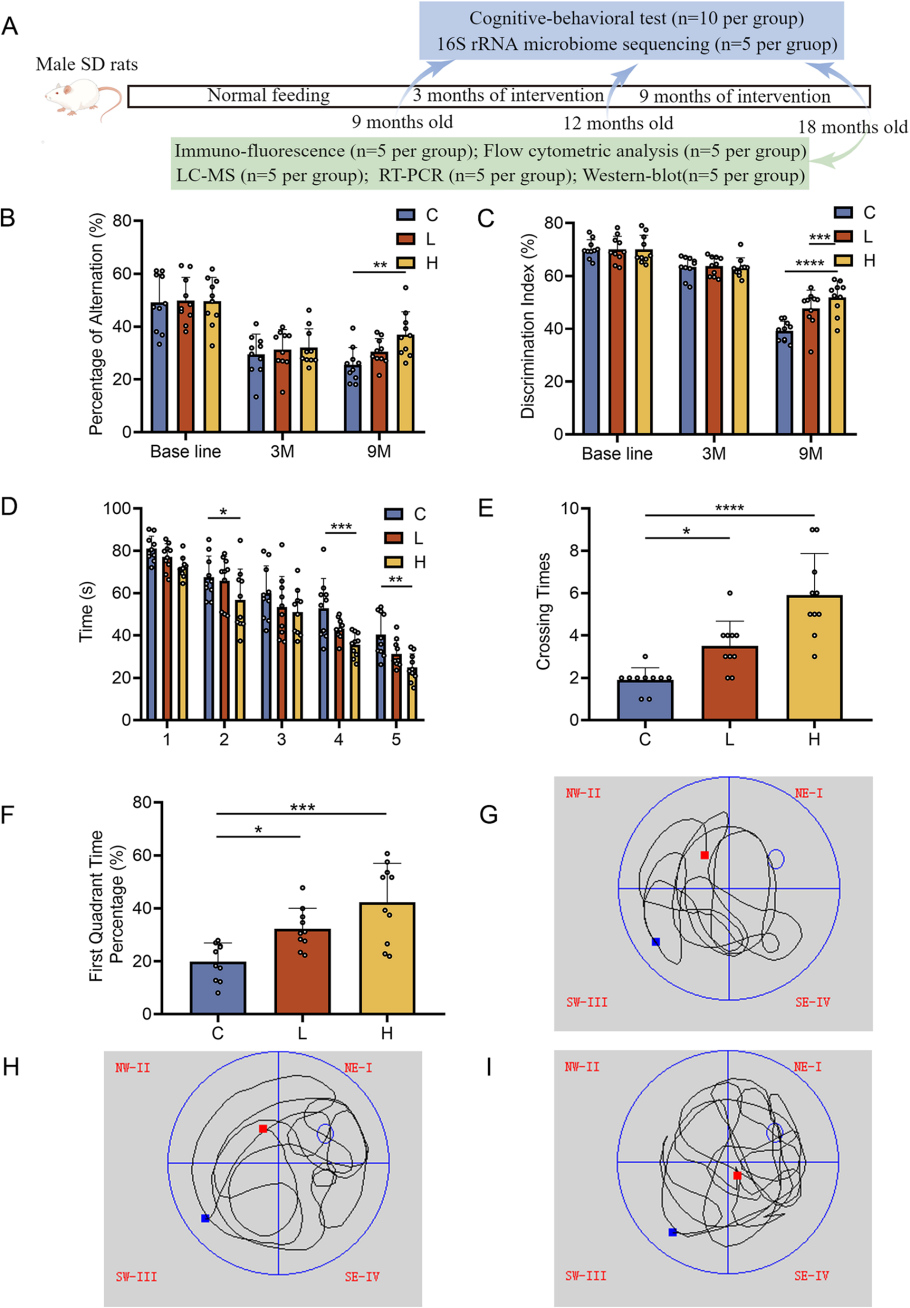
Long-term atorvastatin intervention improves cognitive function in naturally ageing rats (A) Brief experimental procedure and use of rats (B) Y-maze experiment testing the spontaneous rotation behaviour (SRB) score of three groups (*n* = 10 per group). (C) Novel object recognition experiment testing the recognition index (RI) of three groups (*n* = 10 per group). (D) Morris water maze, part one: a five-day positioning cruise experiment in three groups (*n* = 10 per group). (E and F) Morris water maze, part two: space exploration experiment testing crossing times and first quadrant dwell time of three groups (*n* = 10 per group). (G-I) Morris water maze experiment recording the swimming trajectory graphs of three groups (*n* = 10 per group). Data are presented as the mean ± SEM. Statistical analyses were performed using a repeated-measure ANOVA with Tukey’s multiple comparisons test (B, C, D) or a one-way ANOVA with Tukey’s multiple comparison test (E and F). *P < 0.05, **P < 0.01, ***P < 0.001, ****P < 0.0001. C: control group, L: low-dose atorvastatin group, H: high-dose atorvastatin group

To test whether long-term atorvastatin could improve spatial memory decline in naturally ageing rats, we performed a Y-maze experiment. The results showed that the percentage of alternation of control rats significantly decreased at 9 months compared to the baseline, which is consistent with the cognitive decline caused by normal ageing. There was no significant difference in the percentage of alternation among groups (Fig. 1B, C vs. L, *P* = 0.786; Fig. 1 B, C vs. H, *P* = 0.632) at 3 months, but the percentage of alternation was significantly improved at 9 months in the high-dose vs. control group (Fig. 1B, *P* < 0.01), suggesting the protective effect of long-term atorvastatin on cognitive function in naturally ageing rats. Although no significant difference was observed in the low-dose vs. control group (Fig. 1B, *P* = 0.2122), the percentage of alternation of the rats in the low-dose group also showed an improved trend (Fig. 1B).

To test whether atorvastatin improves learning memory ability in aged rats, we first performed a novel object recognition test. The results showed a significant decrease in the discrimination index of control rats at 9 months compared to baseline, indicating a gradual decline in learning memory ability. There was no significant difference in the discrimination index among groups at 3 months, but the discrimination index of rats was significantly improved at 9 months in both the low-dose group (Fig. 1C, *P* < 0.001) and the high-dose group (Fig. 1C, *P* < 0.0001) compared to the control group, with more significant improvement in the high-dose group.

The Morris water maze tests were performed at 9 months. The results showed that atorvastatin rats could find the underwater platform faster than control rats, and high-dose atorvastatin rats took less time (Fig. 1D). The spatial exploration experiment starting on Day 6 showed a significant increase in the crossing times of the target platform in the low-dose (Fig. 1E, *P* < 0.05) and high-dose atorvastatin intervention groups (Fig. 1E, *P* < 0.0001) compared to the control group. Regarding the dwell time in the first quadrant, the results showed that the dwell time was significantly higher in both the low-dose atorvastatin intervention group (Fig. 1F, *P* < 0.05) and the high-dose atorvastatin intervention group (Fig. 1F, *P* < 0.0001) than in the control group. To observe the changes in rat behaviour more visually, we recorded the typical swimming trajectories of the three groups of rats (Fig. 1G-I).

In summary, three behavioural experiments demonstrated that long-term high-dose atorvastatin significantly improved cognitive-related behavioural scores in naturally ageing rats.

**Fig. 2:**
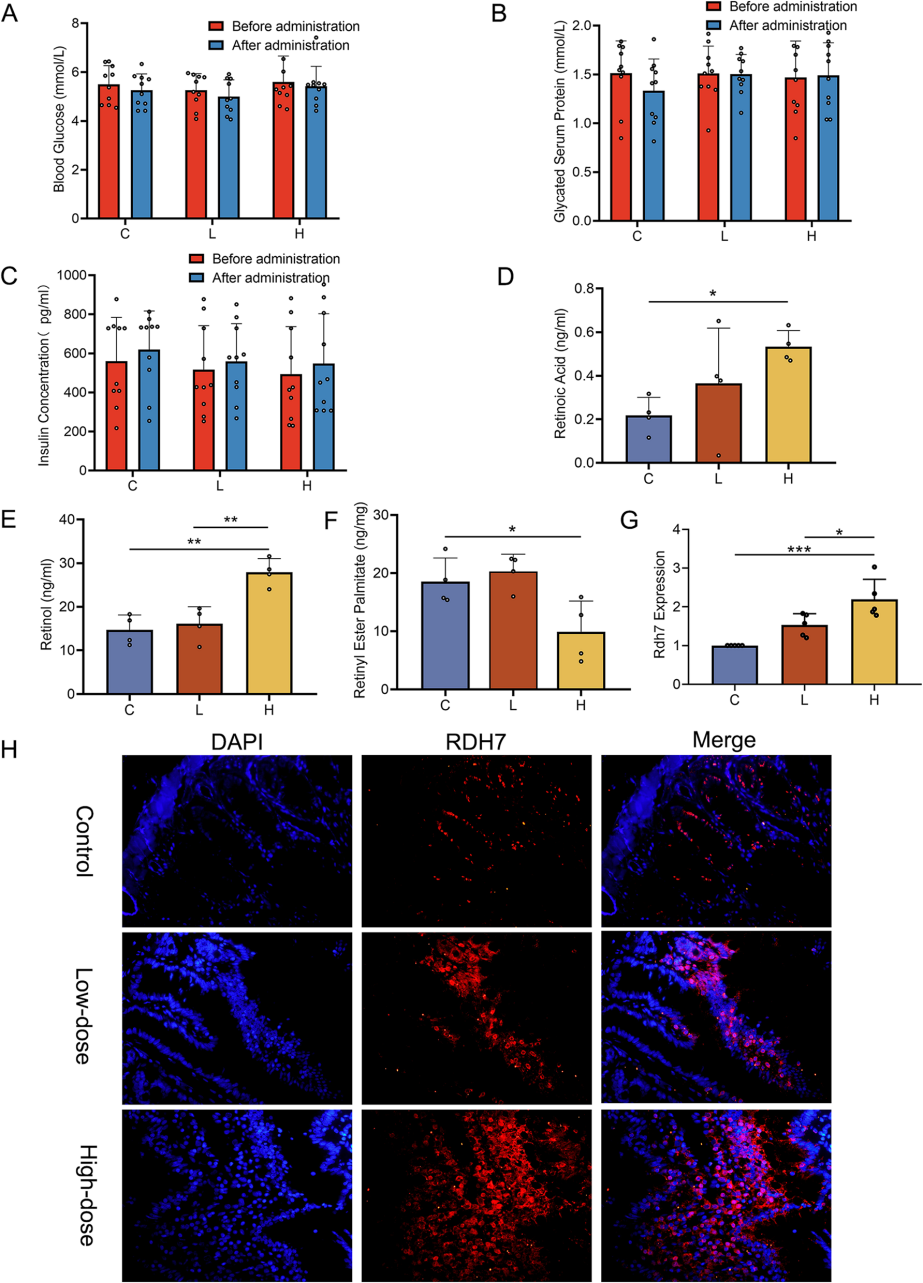
Atorvastatin regulated intestinal retinoic acid (RA) metabolism and storage (A-C) Quantification of blood glucose, glycosylated serum protein and insulin concentrations in the serum of each group of rats (*n* = 10 per group). (D-F) Retinoic acid (RA), retinol (ROH) and retinyl esters (RE) in the small intestine were quantified by LC‒MS in the three groups (*n* = 4 per group). (G) Quantification of Rdh7 mRNA in small intestinal tissues by qPCR in the three groups (*n* = 5 per group). (H) Immunofluorescence of small intestine tissues in the three groups (*n* = 5 per group). Immunofluorescence of Rdh7 (red) in small intestinal tissue; DAPI was used to visualize nuclei (blue). Data are presented as the mean ± SEM. Statistical analyses were performed using a repeated-measure ANOVA with Sidak’s multiple comparisons test (A, B, C) or a one-way ANOVA with Tukey’s multiple comparison test (D, E, F, G). *P < 0.05, **P < 0.01, ***P < 0.001. C: control group, L: low-dose atorvastatin group, H: high-dose atorvastatin group

To test the safety of long-term atorvastatin, related indicators of pancreatic islet function, liver function, and muscle were measured in rats before and after administration of the drug. The results showed that there were no significant differences in blood glucose (Fig. 2A, Group C, before administration vs. after administration, *P* = 0.87; Fig. 2A, Group L, before administration vs. after administration, *P* = 0.84; Fig. 2A, Group H, before administration vs. after administration, *P* = 0.94), glycosylated serum protein (Fig. 2B, Group C, before administration vs. after administration, *P* = 0.50; Fig. 2B, Group L, before administration vs. after administration, *P* > 0.99; Fig. 2B, Group H, before administration vs. after administration, *P* = 0.99) or insulin concentrations (Fig. 2C, Group C, before administration vs. after administration, *P* = 0.91; Fig. 2C, Group L, before administration vs. after administration, *P* = 0.96; Fig. 2C, Group H, before administration vs. after administration, *P* = 0.93) in each group before and after administration.

### Long-term atorvastatin regulated intestinal RA metabolism and storage

To assess whether long-term atorvastatin affects retinoic acid (RA) metabolism, we applied LC‒MS to quantify various vitamin A metabolites in intestinal tissues of rats after 9 months of treatment. The results showed that RA and retinol (ROH) (a precursor form of RA) concentrations were significantly higher in the high-dose atorvastatin group than in the control group (RA, Fig. 2D, *P* < 0.05; ROH, Fig. 2E, *P* < 0.01). There was no significant difference in RA or ROH between the low-dose atorvastatin and control groups (RA, Fig. 2D, *P* = 0.42; ROH, Fig. 2E, P = 0.84). As the storage form of ROH, retinyl esters (RE) were found to be significantly decreased in the high-dose atorvastatin vs. control group (Fig. 2F, *P* < 0.05), but there was no significant difference between the low-dose atorvastatin and control groups (Fig. 2F, *P* = 0.83). These results suggested that long-term high-dose atorvastatin inhibited the conversion of ROH to RE and facilitated the metabolism of ROH to RA, finally resulting in more conversion of ROH to the active form.

### Long-term atorvastatin may regulate intestinal RA metabolism via Rdh7

We detected Rdh7 in the intestinal epithelium of rats using RT‒PCR. The results showed no significant difference between the low-dose atorvastatin and control groups, but the expression level of Rdh7 was significantly higher in the high-dose atorvastatin vs. control group (Fig. 2G, *P* < 0.001).

Although vitamin A in the small intestine can only be absorbed by intestinal epithelial cells (IECS), dendritic cells and stromal cells in the intestine can metabolize vitamin A into RA [30–32]. To investigate the regulatory relationship of RDH7 on RA, we performed immunostaining on rat small intestine tissue sections. The results showed that long-term atorvastatin specifically increased Rdh7 expression in rat IECs compared with that in the control group (Fig. 2H).

These results collectively suggest that long-term atorvastatin may increase RA concentrations in the intestine by regulating the vitamin A metabolism gene Rdh7 in the IECS.

### RA metabolism affected T-cell differentiation to reduce neuroinflammation mediated by the gut-brain axis

**Fig. 3:**
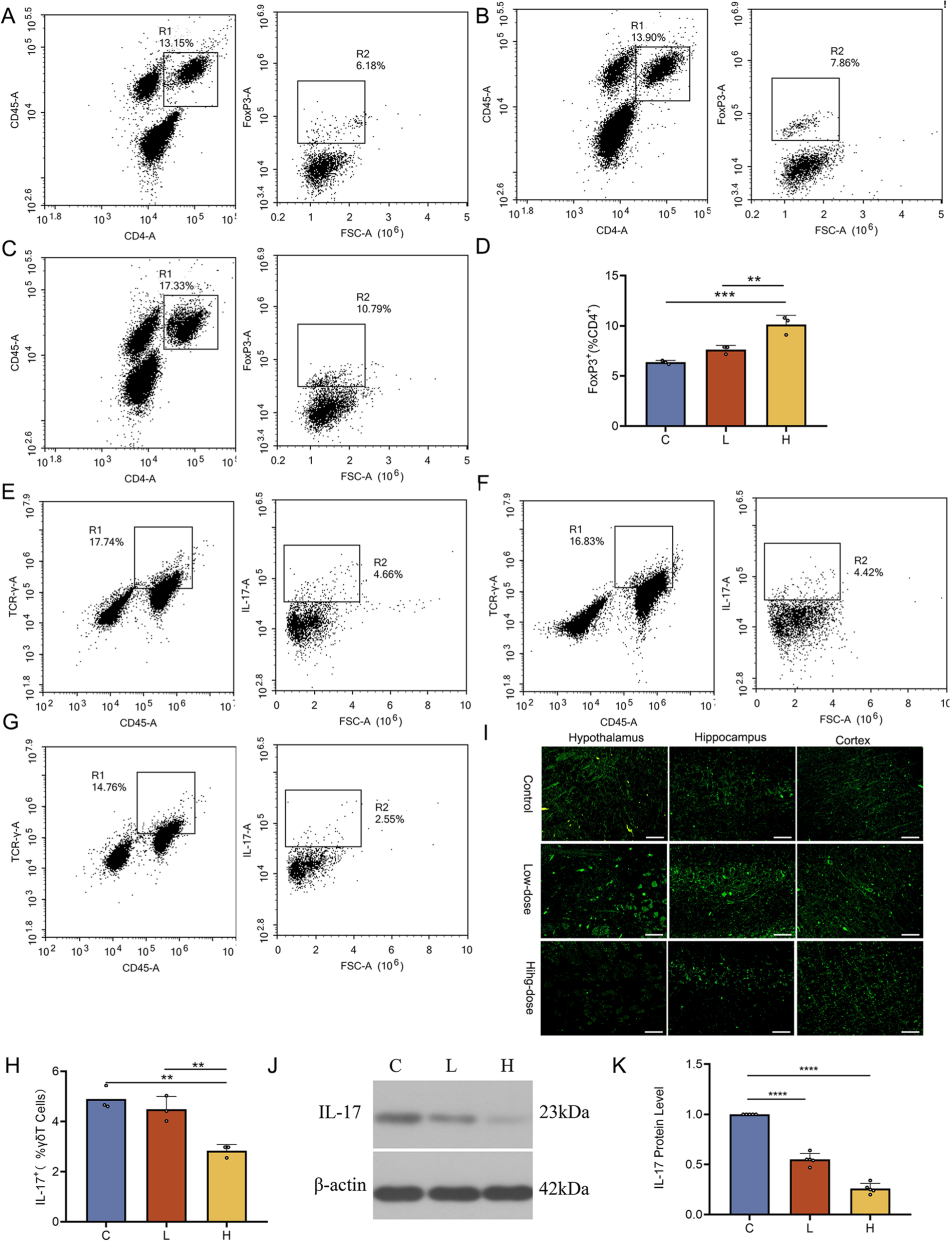
RA metabolism affected T-cell differentiation to reduce neuroinflammation mediated by the gut-brain axis. (A) The number of FoxP3 + cells in the small intestine of the control group (*n* = 3). Representative flow cytometry plots of CD4 T cells identified using CD45^+^ and CD4^+^ expression. Representative flow cytometry plots of T_reg_ cells (CD45^+^CD4^+^FoxP3^+^) in the small intestine [38] (numbers represent events within the gate as a percentage of CD4^+^ cells). (B) The number of FoxP3^+^ cells in the small intestine of the low-dose atorvastatin group (*n* = 3). (C) The number of FoxP3^+^ cells in the small intestine of the high-dose atorvastatin group (*n* = 3). (D) Comparison of the three groups showed that atorvastatin significantly increased the number of FoxP3^+^ cells (*n* = 3 per group). (E) Representative flow cytometry analysis of IL-17 production in γδ T cells (CD45^+^TCR-γδ^+^) in the small intestine of the control group (*n* = 3). (F) Representative flow cytometry analysis of IL-17 production in γδ T cells in the small intestine of the low-dose atorvastatin group (*n* = 3). (G) Representative flow cytometry analysis of IL-17 production in γδ T cells in the small intestine of the high-dose atorvastatin group (*n* = 3). (H) Quantification of IL-17-producing cells in the small intestine of the three groups. (I) Immunofluorescence of the hippocampus, cerebral cortex, and hypothalamus. Immunofluorescence of neurofibrillary tangles (green) in cerebral tissue. (J and K) The expression of IL-17 in the hippocampus of different groups of rats was detected by western blotting (*n* = 3 per group). Data are presented as the mean ± SEM. Statistical analyses were performed using one-way ANOVA with Tukey’s multiple comparison test (D, H, J). **P < 0.01, ***P < 0.001, ****P < 0.0001. C: control group, L: low-dose atorvastatin group, H: high-dose atorvastatin group

Flow cytometric analysis of T_reg_ cells in rat small intestinal tissue showed no significant difference between the low-dose atorvastatin and control groups, but a significant increase was found in the high-dose atorvastatin vs. control group (Fig. 3A-D, *P* < 0.001). Similarly, IL-17^+^γδ T cells showed no significant difference between the low-dose atorvastatin and control groups, but a significant decrease was found in the high-dose atorvastatin vs. control group (Fig. 3E-H, *P* < 0.01). Inflammatory factors can induce Aβ production, phosphorylation of tau, and oxidative stress [33–35]. Thus, inflammation can directly affect the formation of plaques and neurofibrillary tangles, two neuropathological events that in turn can increase inflammation. Neurofibrillary tangles are a characteristic pathological alteration of cognitive decline in rats, and the sites where neurofibrillary tangles generally occur in age-related diseases are the hippocampus, cerebral cortex, and hypothalamus [36, 37]. To explore the changes in different functional brain areas, we performed immunostaining of the hippocampus, cerebral cortex, and hypothalamus. The results showed that long-term high-dose atorvastatin treatment reduced the expression of neurofibrillary tangles at the three sites compared to the control treatment (Fig. 3I). Because the reduction was more pronounced in the hippocampal region and was highly correlated with cognitive function, we only examined the relevant brain tissue in the hippocampal region. Furthermore, quantitative analysis of IL-17 in the hippocampal region tissue by western blot showed that the expression of IL-17 was significantly lower in the low-dose atorvastatin (Fig. 3J-K, *P* < 0.0001) and the high-dose atorvastatin groups (Fig. 3J-K, *P* < 0.0001) than in the control group. Collectively, these results suggest that long-term atorvastatin treatment can affect the ratio of T_reg_ cells to γδ T cells in the small intestinal epithelium, which may affect the cognitive function of aged rats by reducing the transport of IL-17^+^γδ T cells to the meninges via the gut-brain axis, thereby modulating the level of neuroinflammation.

### Long-term atorvastatin increased intestinal flora richness in naturally ageing rats

**Fig. 4:**
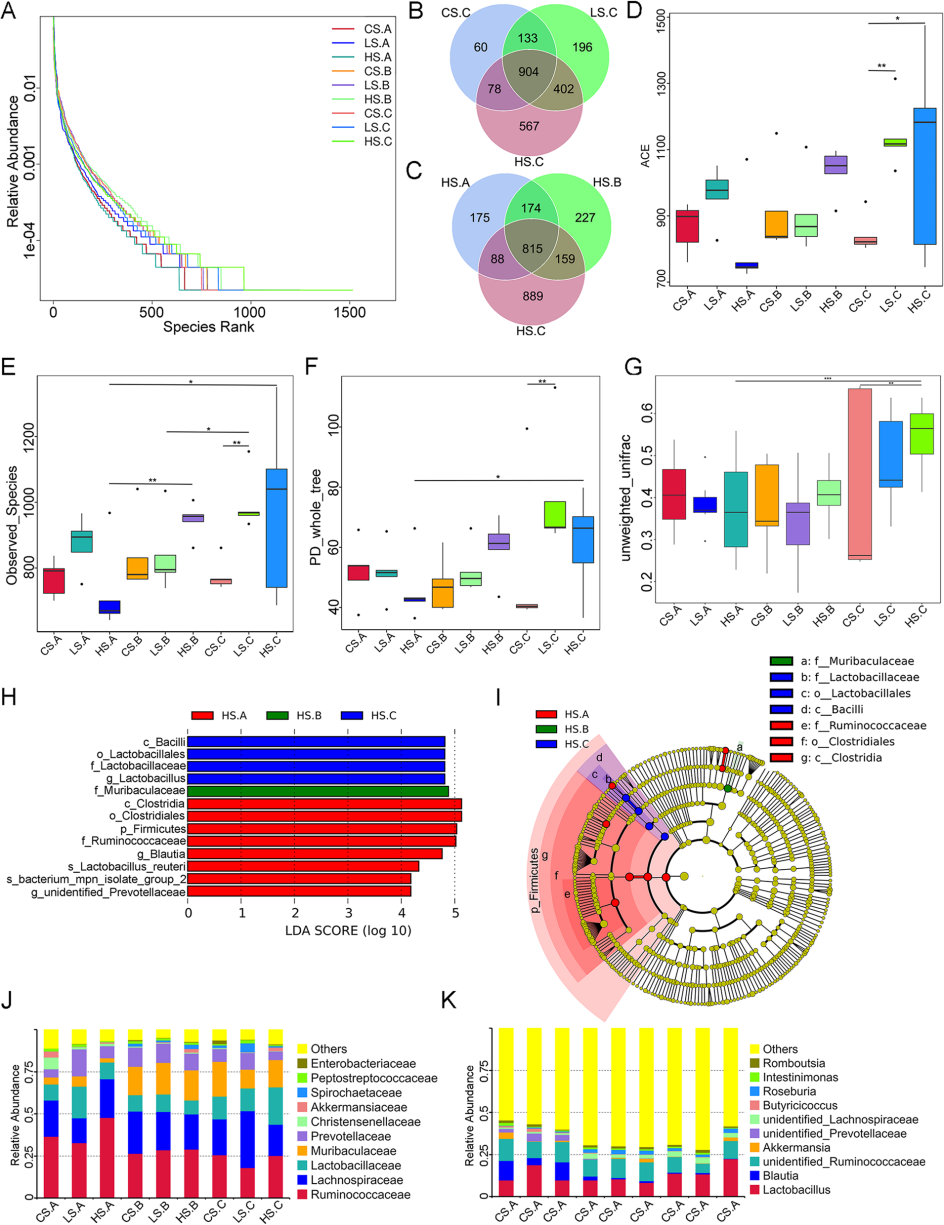
Long-term high-dose atorvastatin increased intestinal flora richness in naturally ageing rats. (A) The rank abundance plot of each group showed that the HS. The C group had the best species richness and the most uniform species distribution. (B and C) The Venn diagram shows the common and unique OTUs among the different groups. (D-F) The microbial alpha diversity differences (ACE index, observed species index, and PD whole tree index) among groups. (G) Analysis of beta diversity (box plot based on unweighted UniFrac beta diversity) among groups. (H and I) The different time points of the high-dose atorvastatin intervention group were analysed by LDA effect size analysis. (J and K) The top ten groups in terms of abundance at the family and genus levels for different groups. Data are presented as the mean ± SEM. Statistical analyses were performed with the Wilcoxon test (D, E, F, G). *P < 0.05, **P < 0.01. CS. A: control group before intervention; CS. B: control group at 3 months; CS. C: control group at 9 months; LS. A: low-dose atorvastatin group before intervention; LS. B: low-dose atorvastatin group at 3 months; LS. C: low-dose atorvastatin group at 9 months; HS. A: high-dose atorvastatin group before intervention; HS. B: high-dose atorvastatin group at 3 months; HS. C: High-dose atorvastatin group at 9 months (*n* = 5 per group)

The rank abundance plot (Fig. 4A) shows the best species richness and the most uniform species distribution at 9 months in the high-dose atorvastatin group, and the species tended to be rich and uniform over time, which suggested that high-dose atorvastatin may improve the richness and uniformity of the intestinal flora. The results of OTUs were obtained based on clustering. The common and unique OTUs between different groups were analysed by plotting them into a Venn diagram. After 9 months of intervention, there was little difference in OTU species unique to the low-dose atorvastatin group compared to the control group, but there was a significant increase in OTU species unique to the high-dose atorvastatin intervention group compared to the control group (Fig. 4B). In the high-dose atorvastatin group, the unique OTU category changed gradually with time, most significantly at 9 months of intervention (Fig. 4C). Alpha diversity analysis also confirmed these findings. The ACE index, observed species index, and PD whole tree index were significantly higher in the long-term high-dose atorvastatin group than in the control group (Fig. 4D-G). In summary, long-term high-dose atorvastatin can increase the richness and diversity of bacterial flora.

Box plots of the beta diversity intergroup difference analysis showed that the flora within the long-term high-dose atorvastatin group was significantly different from the remaining groups. First, long-term high-dose atorvastatin intervention reshaped the microbiota of ageing rats. Second, the LDA effect size analysis (Fig. 4H-I) showed that the significant differences in the intestinal flora of rats at each level were Clostridia, Clostridiales and Ruminococcaceae at baseline, while the significantly different bacteria at each level were Muribaculaceae at 3 months and Lactobacillaceae, Lactobacillale, and Bacilli at 9 months after high-dose atorvastatin treatment. In addition, we found significant differences in the composition of intestinal flora among the groups at the family and genus levels (Fig. 4J-K). Among the top ten groups ranked in abundance at the genus level, significantly higher levels of Lactobacillus and downregulated abundance of Blautia were identified at 9 months in the high-dose atorvastatin vs. control group. Among the top ten flora ranked in abundance at the family level, significantly higher levels of Lactobacillus and downregulated abundance of Ruminococcaceae were observed at 9 months in the high-dose atorvastatin vs. the control group. These results may indicate that the abundance of probiotic flora in the intestine can be increased after long-term high-dose atorvastatin intervention.

## Discussion

In the current study, we identified several findings in naturally ageing rats: (1) long-term atorvastatin improved cognitive decline; (2) long-term atorvastatin can affect RA metabolism by altering Rdh7 expression in the intestine, which in turn leads to the proliferation of intestinal T_reg_ cells and inhibition of IL-17^+^γδ T-cell function; (3) long-term atorvastatin decreased IL-17 expression in the hippocampal region tissue; and (4) long-term atorvastatin increased intestinal flora richness. Collectively, these findings provide the first evidence that long-term atorvastatin intervention prevents cognitive decline in naturally ageing rats through the gut-brain axis.

The absorption and metabolism of vitamin A by IECs take place in close association with microorganisms and immune cells. Vitamin A is an essential fat-soluble nutrient that plays a key role in various metabolic and physiological processes in the body [39, 40]. Animals cannot synthesize vitamin A on their own but can obtain it through diet. IECs are the main cells that absorb dietary vitamin A. After vitamin A uptake by IECs, vitamin A can be further metabolized to retinyl esters (RE) for storage in the liver or to retinoic acid (RA) in a two-step process [41]. We found that long-term high-dose atorvastatin inhibited the conversion of ROH to RE and promoted the metabolism of ROH to RA. Rdh7, a gene expressed only in the intestine, has been found to catalyse the oxidation of ROH to retinaldehyde, which in turn mediates the conversion of retinaldehyde to RA via retinaldehyde dehydrogenase (Raldh) [7]. Our study found a significant increase in RDH7 expression after long-term atorvastatin intervention. Based on these results, we infer that the elevated RA concentration induced by atorvastatin may be due to the upregulation of Rdh7 expression.

RA has an important role in the regulation of immune cell differentiation and promotes the differentiation of naive T cells into T_reg_ cells. A recent study found that gut flora stimulation led to the expansion of small intestinal T_reg_ cells and suppressed the function of IL-17^+^γδ T cells, which were transported from the gut to the brain, where they localized to the meninges and enhanced neuroinflammation by secreting IL-17 [38]. γδ T cells were found to be a major source of IL-17 in healthy meninges and increase meningeal IL-17 levels to exacerbate synaptic dysfunction, which underlined the early cognitive decline in AD [42]. In addition, it has also been shown that IL-17 neutralizing antibody (IL-17Ab) can reduce amyloid‐β (Aβ)‐induced neuroinflammation [43]. In the current study, we found that long-term high-dose statins upregulated small intestinal T_reg_ cells, inhibited small intestinal IL-17^+^γδ T cells and decreased IL-17 expression in the hippocampus of the brain. These results suggest that long-term atorvastatin may attenuate neuroinflammation caused by IL-17^+^γδT cells via the gut-brain axis by modulating intestinal RA metabolism, which in turn improves cognitive function.

The interaction between intestinal flora and the IECS is crucial for the immune response [32]. The intestinal lamina propria and epithelium contain the largest number of T cells in the body, especially γδ T cells, and the intestinal microbiota provides different signals to the host immune system. In a healthy state, more than 80% of the intestinal flora consists of specialized anaerobic bacteria belonging to the phylum Thick-walled Bacteria and Bacteroidetes [44]. Ageing decreases both the diversity and stability of the intestinal flora. For example, the number of Enterobacteriaceae and Enterococcus in the gut increased significantly in the older population compared to the younger population, while the number of Bifidobacteria decreased [45]. Dysbiosis of the gut microbiota can lead to dysregulation of the gut-brain axis and cause a neuroinflammatory response in the brain, which in turn affects cognitive function [46]. Previous studies have found that atorvastatin in the treatment of hypercholesterolaemic rats not only lowered their cholesterol levels but also increased the abundance of intestinal flora and improved the gut microbial composition [47]. In the current study, we found that the significantly different bacteria in control rats were class Clostridia compared to atorvastatin intervention rats. A study found that commensal bacteria belonging to the class Clostridia regulated the RA concentration in the intestine by suppressing the expression of RDH7 in IECs [7]. Taken together, the current results suggest that upregulation of Rdh7 by atorvastatin may be achieved by inhibition of Clostridia, but the exact mechanism of regulation needs to be verified by further studies. Furthermore, we found a significant increase in intestinal flora richness and probiotic (mainly Lactobacillale) abundance in aged rats after long-term high-dose atorvastatin. A recent study found that intake of probiotics significantly improved memory deficits in aged SAMP8 mice by improving the composition of the intestinal and brain flora and inhibiting inflammatory factors in the brain and intestine [48]. Similarly, another study reported that intake of specific probiotics effectively prevented and improved AD through the gut-brain axis [49]. In agreement with these studies, we also found that changes in intestinal flora richness had the same trend as changes in cognitive function, which may further suggest that the improvement of cognitive function by atorvastatin is closely related to the intestinal flora.

The level of fungal flora is also closely related to cognitive function. Intestinal fungal communities maintain the balance of the intestinal environment, influence the inflammatory response, and regulate immune function [50, 51]. One study found that intestinal colonization of specific mucosa-associated fungi in the mouse intestine induced systemic IL-17A production and promoted social behaviour in mice by acting on neurons [52]. Another study found that mild cognitive impairment patients contained more of a specific type of fungus and less of other fungi compared to healthy controls. Specific fungi in the gut may be directly associated with an increased risk of developing AD [53]. Intestinal fungal disorders are closely related to AD, but the underlying mechanisms have not been fully discovered [54, 55]. We intend to investigate the effects of long-term atorvastatin treatment on intestinal fungi and cognitive function in the next study.

A meta-analysis of statin RCTs showed a 9% increase in the incidence of new-onset diabetes treated with statins [56]. Our results showed that after long-term statin treatment, there was no significant change in glucose-related indicators in either the low-dose or high-dose groups. This suggests that long-term high-dose statins have a good safety profile in terms of diabetes risk.

**Fig. 5:**
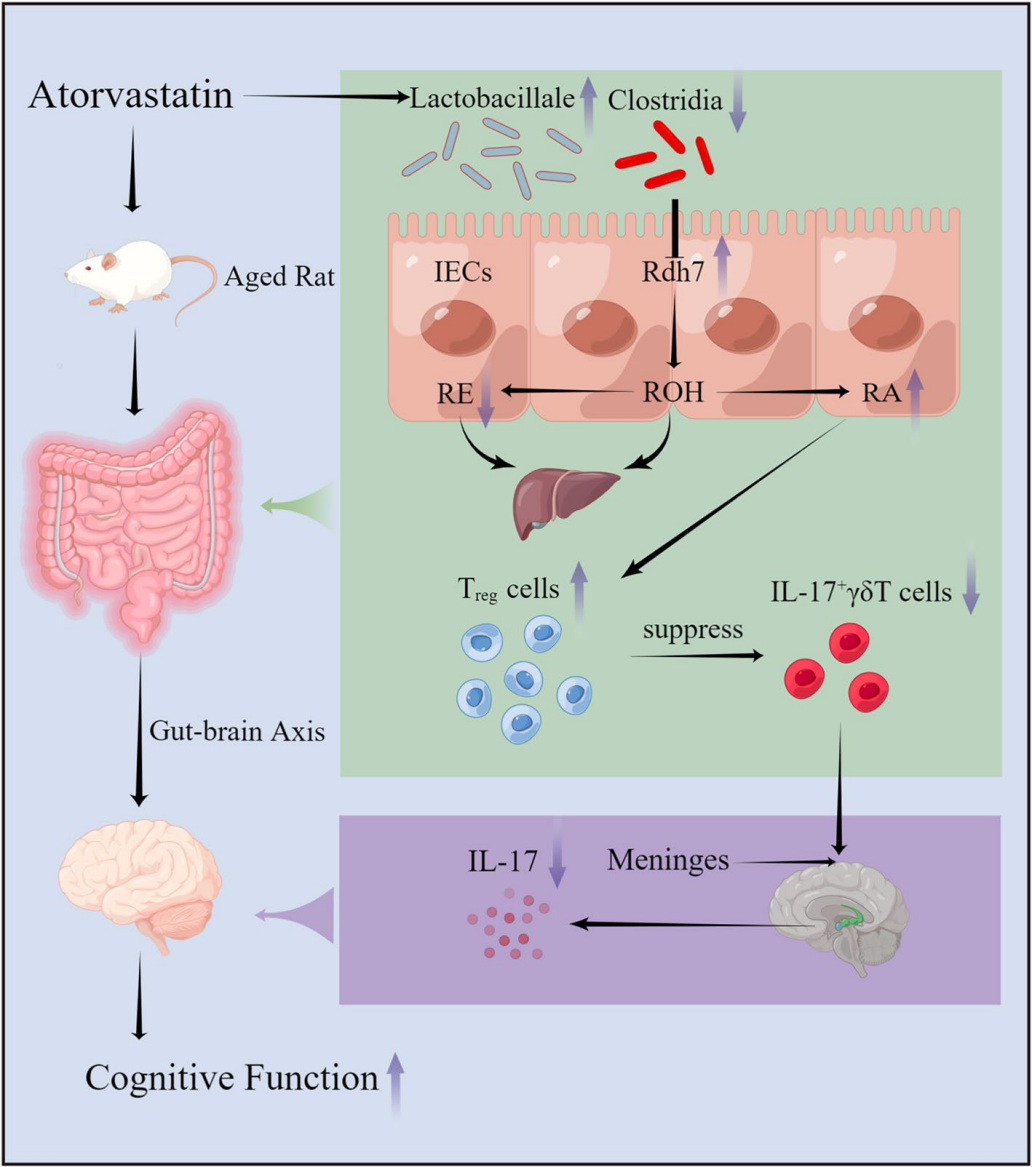
Potential mechanisms underlying the effect of long-term atorvastatin on cognitive impairment in aged rats

The main strength of this study is that it is the first study to investigate the effect of long-term statins on cognitive decline in naturally ageing rats, which will provide important information regarding the clinical debate about the effect of long-term statins on cognition. In addition, the current study presented a new mechanism underlying the effect of long-term atorvastatin treatment on cognitive impairment in naturally ageing rats (Fig. 5). First, long-term high-dose atorvastatin increased the abundance and diversity of intestinal flora, with a significantly higher probiotic abundance and a lower abundance of Clostridium perfringens, which may affect RA metabolism by altering Rdh7 expression in the intestine. In turn, enhanced RA metabolism may lead to the proliferation of intestinal Treg cells and inhibition of IL-17^+^γδ T-cell function. Second, the inhibition of intestinal IL-17^+^γδ T cells may reduce their transition through the gut-brain axis to the meninges [38], which will further reduce IL-17 secretion in the hippocampal region of brain tissue to inhibit neuroinflammation. Finally, these changes improve cognitive decline in aged rats.

## Conclusion

In summary, this is the first report that long-term atorvastatin can safely improve cognitive decline associated with natural ageing in rats via the gut-brain axis. The findings will provide a new therapeutic target for antiaging and improving cognitive function.

## Limitations of the study

We must acknowledge that this study has several limitations. First, only male rats were used in our study, while the effects of atorvastatin on female rats should be investigated in future studies because disturbances in the gut microbiota were found to affect the hallmark pathological features of male, but not female, AD mice [57]. Second, in the current study, we did not investigate the link between the improvement of intestinal flora richness by atorvastatin and the regulation of RA in naturally ageing rats. We intend to explore this further using faecal microbiota transplantation in germ-free conditions. Third, inflammation is influenced by a variety of factors, and our study has currently been conducted on the small intestine, and other intestinal regions were not systematically studied. We will next investigate unique sets of microbiomes to other intestinal regions (particularly the colon).

## Data Availability

Requests for data collected for the study can be made to the corresponding authors and will be considered on reasonable request.

## Abbreviations

AD: Alzheimer’s disease
RA: Retinoic acid
RE: Retinyl esters
ROH: Retinol
LDL-C: Low-density lipoprotein cholesterol
Rdh7: Retinol dehydrogenase 7
IECS: Intestinal epithelial cells
Raldh: Retinaldehyde dehydrogenase
Aβ: Amyloid-β peptide
IL-17Ab: IL-17 neutralizing antibody
